# Football, National Quarantine and the Governor’s Veto

**Published:** 1898-01

**Authors:** 


### FOOTBALL, NATIONAL QUARANTINE AND THE GOVERNOR'S VETO.

The Georgia General Assembly has adjourned and, as usual, all sorts of bills, good, bad, and indifferent, have been enacted into laws.

Some of the measures which were considered in this session of the legislature were of some interest to the medical profession. In the first place we congratulate our cousins, the dentists, upon securing the law regulating the practice of dentistry. It was much needed, and we hope the results will be salutary and far-reaching.

The bill prohibiting intercollegiate match games of football, which passed both houses almost unanimously, was coolly vetoed by the Governor, upon the ground that it was "legislation which seeks to usurp the responsibility and functions of trustee, faculty, and parent, and take charge of young men and children in their sports; it is governmental paternalism of the most vicious and pronounced type; [and therefore] fundamentally wrong." The

Governor unfortunately admitted that "some legislation may be necessary to modify the fierceness of football games"; upon which the *Medical Record* appropriately remarks that the Governor "seemingly overlooked the fact that there is just as much paternalism in deciding upon the exact amount of violence permissible in a game as there is in prohibiting it." The necessity for suppressing some of the "fierceness" and "violence" of the modern game of football seems to be admitted. To what extent our long-haired, padded and pneumatic ball-warriors of the future will be allowed to indulge their tastes in this direction must now be determined, we suppose, by "trustee, faculty and parent." We understand that a meeting of the Southern Intercollegiate Athletic Association was lately held in Birmingham, for the sole purpose of modifying the rules of the game. We trust that the modifications will be radical.

The legislature also passed a bill recommending that quarantine regulations be placed under national control. The Governor vetoed that also ; partly on the ground that the bill, as passed, could have no weight as law, principally on the ground that it was a surrender of the State's rights to the government. Suppose it is. If the government can do what the States cannot or will not do, why not give it the privilege? State quarantine has time and again demonstrated its inability to deal with yellow fever. While our esteemed Governors are seeking to maintain the dignity of "State's rights" hundreds of lives and millions of commerce may be swept away. The national government, through an efficient Marine Hospital Service or Department of Health, is better prepared than any State can be to protect our coasts from foreign-born epidemic diseases. And we believe that the sooner this is conceded, and the sooner medical officers of the government can undertake these duties without hindrance or interference, the sooner we may expect to see the last of yellow fever, and the better it will be for all concerned.

